# The Rotunda and Coombe Hospitals

**Published:** 1917-10-13

**Authors:** 


### MIDWIFERY TEACHING IN DUBLIN.

### The Rotunda and Coombe Hospitals.

From the establishment of the Rotunda Hospital in 1745—or rather of what is now the Rotunda Hospital, for the first institution was in a three-storey house used by a teacher of dancing—up till the present day, Dublin has always taken the lead in matters gynæcological.

The reason for this is not easy to discover; in no other branch of medicine or surgery have the Irish schools established any pre-eminence, but in this limited though essential field their supremacy is indisputable. In a well-known guide to American medical men coming to Europe for the purposes of study, Dublin is set down as the only place in which to learn midwifery, and though Vienna and Viennese methods may, before the war at all events, have had their adherents, the reason was probably that the students there saw many more operations, applications of forceps, etc., and this in cases where the Dublin school have always wisely preferred to leave the case to Nature, rather than have needless recourse to the surgeon's knife. "What are the indications for Cæsarean section?" ran an old students' quip at Vienna. "There is only one," was the answer, "there has not been a case for two days."

### Coombe Lying-in Hospital.

The lying-in hospitals of Dublin are two in number: the Rotunda, whose name is known wherever the art of medicine is practised, and the less known but not less admirable Coombe Hospital, which has of recent years made great advances, although severely handicapped by insufficient funds and cramped accommodation. Let us first pay it a visit, reserving its more famous sister for later consideration. Situated as it is in one of the poorest districts of Dublin, in one of the most malodorous of that city's many noisome slums, it naturally supplies a great and crying need. The Irish are the most prolific race in Europe, and the infantile rate of mortality in Dublin, especially during the summer months, is an appallingly high one. Little is done by the municipal authorities for the improvement of the one-room tenements and underground hovels in which the poor are forced to live.

Drunkenness is rife. Girls marry at sixteen, and from that time to the climacteric it is no uncommon thing for a child to appear every year. Contraconceptual methods of any kind are unknown. The demands, therefore, upon the resources of the hospital are very great, and lack of funds imposes serious limitations upon the work it is able to perform.

### Modern Operating Theatres.

The hospital itself is divided into two blocks: one, the larger, being devoted to maternity work, and the other and more recent part to gynæcological work. Both have modern and perfectly equipped operating theatres, dating from 1903, whilst the whole hospital was rebuilt in 1875, thus differing from the Rotunda, where old and insanitary buildings have to be contended against. In addition there is a large out-patient department, in which patients are examined on three days in the week, the gift of the Countess of Pembroke. The accommodation for students, whilst not luxurious, is adequate, and any number up to forty can be taken at one time. Lady students can also enter into residence, a feature in which the Irish hospitals differ from those in this country.

The teaching at any hospital must naturally depend very largely upon the capabilities and enthusiasm of those to whom it is entrusted; and the Coombe Hospital is indeed fortunate in having the services of Dr. R. A. MacLaverty, F.R.C.P., as master. Coupling as he does a rare knowledge of his subject in all its details, with an unusual eloquence and force of delivery, the student who fails to master and to carry away a real enthusiasm for midwifery must be a dullard indeed. The duties of assistant-master are ably carried out by Dr. Morris, M.B., M.A.O., whilst the appointments of clinical clerk (a post corresponding to a house-surgeonship) are eagerly sought after by medical men anxious for further experience in obstetrical work.

### Course of Study.

The course, which may be taken for any length of time, comprises lectures and demonstrations in elementary midwifery by the master and assistant-master, operations on every afternoon in the week, and a demonstration of cases in the out-patient department three times weekly. In addition, resident students attend confinement cases in the district, pairs working together, a regulation adding considerably to the amenities of long hours of waiting. The L.M. diploma of the hospital is granted to students who have attended for six months and have passed the qualifying examination. Altogether, when the small size of the hospital—there are only seventy beds—and the serious financial disability under which it works, are taken into consideration, it shows a record of progressive and conscientious work not easily surpassed.

### The Rotunda Hospital.

This hospital, working as it does on a more ambitious scale, is naturally better known to the generality of medical men.

The "Rotunda treatment" of such conditions as post-partum hæmorrhage and eclampsia is known wherever midwifery is practised, and the insistence of those responsible for its teaching upon the importance of aseptic methods during and after delivery has done much to reduce the mortality from puerperal fever.

The buildings are infinitely more spacious than those of the Coombe, but owing to their great age much additional labour is required to keep the wards free from dust and dirt. Many additions have, however, been made to the original structure, notably in the construction of a distinct gynæcological wing in 1895, and of two beautifully equipped labour wards and theatres in 1912, so that there is a happy combination of modernity and the spacious Georgian architecture of which Dublin has so many fine examples.

A very large number of patients are dealt with by the hospital. Thus in 1912-13, 2,124 patients were confined in their own homes under the care of students attached thereto, and 2,012 were confined in the wards of the hospital; 585 patients were, in addition, admitted to the gnyæcological wards of the hospital.

### Teaching under War Conditions.

The daily routine for students consists of attendance at the morning lectures on midwifery and gynæcology, attendance at and examination of patients in the gynæcological department, attendance at operations, personal conduction of labour cases in the intern and extern maternities, and attendance at all cases of abnormal labour in the wards. The work of the hospital at the moment is unfortunately in a somewhat disorganised state, owing to the absence of the master, Dr. Jellett, on Red Cross service in France, and though his place is being filled by three of the former masters, each for one month in rotation, such an arrangement causes a certain unavoidable lack of continuity in the teaching and routine of the hospital. For this reason criticism must be withheld till an occasion presents of seeing the work under more normal conditions. Lack of funds is not, as with the Coombe, a matter of supreme urgency, as the foundation has had in the past many generous benefactors. In the early nineteenth century the Rotunda Rooms, from which the hospital derives its name, were the centre of social life in Dublin, and concerts and entertainments were run by the governors as a source of revenue. Though long separated financially, the handsome hall with its beautiful Wedgwood frieze makes a charming adjunct to the handsome frontage of the hospital. The war has changed many things, but there can be little doubt that the return of peace will see students and practitioners of all nationalities coming to Dublin in the same numbers and with the same enthusiasm for the value of its teaching as before.

